# Identification of Episomal Human Papillomavirus and Other DNA Viruses in Cytological Anal Samples of HIV-Uninfected Men Who Have Sex with Men

**DOI:** 10.1371/journal.pone.0072228

**Published:** 2013-08-12

**Authors:** Maria Gabriella Donà, Francesca Paolini, Maria Benevolo, Amina Vocaturo, Alessandra Latini, Amalia Giglio, Aldo Venuti, Massimo Giuliani

**Affiliations:** 1 Sexually Transmitted Infection (STI) Unit, San Gallicano Dermatological Institute, Rome, Italy; 2 Virology Laboratory and HPV Unit, Regina Elena National Cancer Institute, Rome, Italy; 3 Pathology Department, Regina Elena National Cancer Institute, Rome, Italy; 4 Microbiology and Clinical Pathology Department, San Gallicano Dermatological Institute, Rome, Italy; Columbia University, United States of America

## Abstract

To date, there have been only few studies that investigated integration of anal Human Papillomavirus (HPV). Most of them were conducted on HIV-infected individuals and mainly analyzed samples from high-grade lesions and invasive cancer. We aimed to investigate HPV physical status in HIV-negative men who have sex with men (MSM) with a detectable anal HPV infection, irrespective of the presence of lesions. We also sought to explore the presence of other circular DNA viruses in the anal region. Study participants were attendees of an STI screening program, which were also screened for anal HPV infection and cytological abnormalities. HPV physical status was assessed using multiply-primed RCA. HPV16-positive samples were also analyzed using E2/E6 multiplex PCR, qRT-PCR and APOT assay. RCA and virus-specific PCR were employed to investigate the presence of other DNA viruses. Anal HPV infection was detected in 76.9% of the 230 MSM enrolled. The anal cytological reports were: 129 NILM, 37 ASC-US and 28 L-SIL (36 samples were inadequate for interpretation). HPV physical status was evaluated in the 109 anal specimens that harbored one or two different HPV genotypes. Integration was observed only in one HPV16-positive sample (0.9%), in which integrate-derived viral transcripts of type B were detected. Integration occurred in chromosome 14 q. In 22 of the 53 (41.5%) mucosal HPV-negative samples, RCA restriction results would seem to indicate the presence of circular DNA viruses. Indeed, cutaneous HPV (4 samples), MCPyV (5 samples) and TTV (4 samples) were detected. In conclusion, anal HPV integration was rarely evidenced in HIV-uninfected MSM with no or mild anal cytological abnormalities, although the integration rate may have been underestimated because of the limitations of the employed assays. Other DNA viruses were detected in the anal samples of these individuals, although the significance of this occurrence needs to be assessed.

## Introduction

Human Papillomavirus (HPV) is responsible for 90–99% of cervical cancer [1]–[3] and 70–100% of anal cancer cases [4], [5]. Only a subset of HPV genotypes (High-Risk) are carcinogenic, such as HPV16 and 18, which represent those most frequently detected in genital cancers [4], [6]. Anal HPV infection is very common in HIV-infected and -uninfected men who have sex with men (MSM), among which anal cancer incidence has been greatly increasing during the last decades [7].

HPV-driven mechanisms of cervical carcinogenesis are largely known. E6 and E7 viral oncoproteins, de-regulating cellular proliferation and inducing genetic instability, are the main responsible for the neoplastic process [8]. A key event seems to be the integration of the viral DNA into the host genome [9], which results in the loss of E2, involved in a negative feedback that controls E6/E7 expression, and affects host gene expression and/or functionality [10]. However, the significance of the integration is not fully understood. In fact, viral integration has been also found in women with no or low-grade cervical intraepithelial neoplasia [11], [12], although integrated forms are more frequent in high-grade lesions and cancer [13].

At present, the available data mainly concern HPV physical status in cervical samples, while only a limited number of studies have ascertained HPV integration in anal infections. In addition, most of them have been conducted in HIV-infected individuals.

The main aim of this study was to analyze HPV physical status in HIV-uninfected MSM with anal infection, irrespective of the presence of lesions. Moreover, we aimed to investigate the anal samples for the presence of other DNA viruses.

## Materials and Methods

### Ethics Statement

The study was approved by the San Gallicano Dermatological Institute Ethics Committee (Prot. CE/564/11) and performed in compliance with the Helsinki Declaration. A written informed consent was obtained from all participants.

### Study population

Study participants were selected among the HIV-uninfected MSM attending the STI Unit of the San Gallicano Dermatological Institute (Rome, Italy) for a large STI-HIV screening program (COROH project), which includes evaluation for anal HPV infections and cytological abnormalities [14], [15].

### Anal sample collection and cytology

A sterile Dracon swab was used to collect anal epithelial cells, then dispersed in PreservCyt (Hologic Inc., Pomezia, Italy) [15]. Cytological slides were obtained using a ThinPrep 2000 processor (Hologic) and independently interpreted by two cytopathologists (MB and AV), blinded to the HPV test results. Cytology was classified following the Bethesda 2001 guidelines, also accepted for the anal district [16].

### Mucosal HPV detection and genotyping

Mucosal HPV were detected and genotyped utilizing the Linear Array HPV Genotyping Test (Roche Diagnostics, Milan, Italy) according to the manufacturer's instructions. HPV genotype risk classification has been specified elsewhere [14].

### Multiply primed Rolling Circle Amplification (RCA)

RCA was performed using the Illustra TempliPhi100 Amplification Kit (GE Healthcare, Milan, Italy) following the manufacturer's instructions with 450 µM extra dNTPs. To resolve the concatemers, RCA products were digested with BamHI, EcoRI or HindIII (Invitrogen-Life Technologies, Monza, Italy), according to the HPV genotype/s present in the samples. Digestion was performed for 3 h at 37°C (EcoRI, HindIII) or 30°C (BamHI) in a total volume of 20 µl. Restriction results were verified by 0.8% agarose gel electrophoresis.

### HPV16 E6/E2 Multiplex PCR

A multiplex PCR for the simultaneous amplification of HPV16 E2 and E6 was performed as described [17]. Amplification products resolved on agarose gel were quantified by densitometric scanning with MFS-6000CX Apparatus (Mustek Systems, Inc., Venlo, The Netherlands). Data were analyzed with Phoretix 1D analysis software (Nonlinear Dynamics, Jahnsdorf Germany) and the relative ratio of E2/E6 PCR products was calculated. As a preliminary standardization, E2/E6 ratio of 20, 10, and 0.5 pg of an HPV16 recombinant plasmid was calculated to set the value of a pure episomal form, as already reported [18]. The cut-off for mixed forms (episomal and integrated genomes), calculated as the mean value minus 2 standard deviations, was equal to 1.53. Samples with E2/E6 ratio ≤1.53 were considered harbouring mixed forms. In our experimental conditions, the sensitivity of multiplex PCR is one integrated sequence (linearized sequence without E2) over 1,000 episomes [17].

### Quantitative Real-Time PCR (qRT-PCR)

SYBR Green-based quantitative Real time PCR was performed using the iCycler iQ detection system (BioRad Laboratories Inc., Milan, Italy), according to the procedures and primers described by Nagao et al [19]. β-actin was used as a normalization gene. Briefly, amplification of HPV16 E2, E6 and β-actin was performed in a total volume of 20 µl containing Kapa SYBR Fast 2× qPCR Master Mix (KAPAbiosystems, Milano, Italy), 10 µM of each specific primer and 20 ng of template. After an initial denaturation at 95°C for 3 min, the following protocol was used for 40 cycles: denaturation at 95°C for 3 s, annealing and extension at 60°C for 30 s. To discriminate among episomal, mixed and integrated viral DNA, an experimental cut-off value was established mixing an E6 plasmid DNA with full-length HPV-16 DNA in different copy numbers, in order to represent pure episomal (full-length exclusively), pure integrated (E6 exclusively), and mixed forms (20 to 80% integrated). Statistical analysis demonstrated significant differences between copy number for E2 and E6 when sample DNA contained an excess of 40% E6 plasmid DNA in solution, as already reported by Nagao et al [19]. The cut-off value for the ratio of E2 to E6 copy numbers to distinguish mixed from pure episomal forms was set at 0.60.

### Detection of HPV16 E7 transcripts and Amplification of Papillomavirus Oncogene Transcript (APOT) assay

Total nucleic acids were treated with DNase (Invitrogen) for 15 min at room temperature and, after EDTA addition, 10 min at 65°C. Total RNA was reverse transcribed using the Advantage RT for PCR kit (Clontech, Jesi, Italy) and an oligo(dT)17 primer coupled to a linker sequence (p3) [20]. RNA integrity and first-strand cDNA quality were verified using β-actin primers [21]. cDNA encompassing E7 oncogene sequences was amplified using HPV16 E7-specific primers, as described previously [22]. The APOT assay was then performed using HPV16 E7-specific forward primers and (dT)17-p3 reverse primer as described by Klaes and collaborators [13]. PCR products were gel-purified after electrophoresis using QIAquick spin columns (QIAquick PCR purification Kit, Qiagen), according to the manufacturer's instructions. Purified fragments were subjected to direct sequencing (Bio-Fab Research, Rome, Italy) and DNA sequences were compared with the reference sequences of the National Center for Biotechnology Information (NCBI) Entrez Nucleotide database, using BLASTN-program.

### Cutaneous HPV detection

Cutaneous HPV DNA was amplified using CP65/70 primer set in the first PCR and CP66/69 in the nested PCR, as described elsewhere [23], [24]. Amplification was verified by agarose gel electrophoresis.

### Merkel Cell Polyomavirus (MCPyV) detection

MCPyV DNA detection was performed using the LT1primer set in the first PCR and M1/M2 in the nested PCR (nPCR), as previously described [21]. Amplicons were visualized by agarose gel electrophoresis. nPCR products were purified and sequenced (Bio-Fab Research, Rome, Italy), using M1 and M2 primers. Sequencing results were compared to all genomic databases using BLASTN-program provided by the NCI.

### Torque Teno Viruses (TTV) detection

The presence of TTV was investigated by PCR using RCA products as a template and the NG054/NG147 primer set in the first PCR and the NG132/NG133 primer set in the nPCR [25], following a previously described protocol [26].

## Results

The first 230 consecutive HIV-uninfected MSM of the COROH project, screened for anal HPV infection and morphological changes, were included in the present study. The median age of these individuals was 32 (IQR 26–39) and 96.9% of them were Caucasian. The other socio-demographic and behavioral characteristics of the study participants were similar to those of the larger cohort, which have been described elsewhere [14] (data not shown).

In regard to anal cytology, 36 samples (15.6%) were inadequate for the evaluation. The cytological reports for the remaining 194 participants were: 129 NILM (66.5%), 37 ASC-US (19.1%) and 28 L-SIL (14.4%).

**Figure 1:**
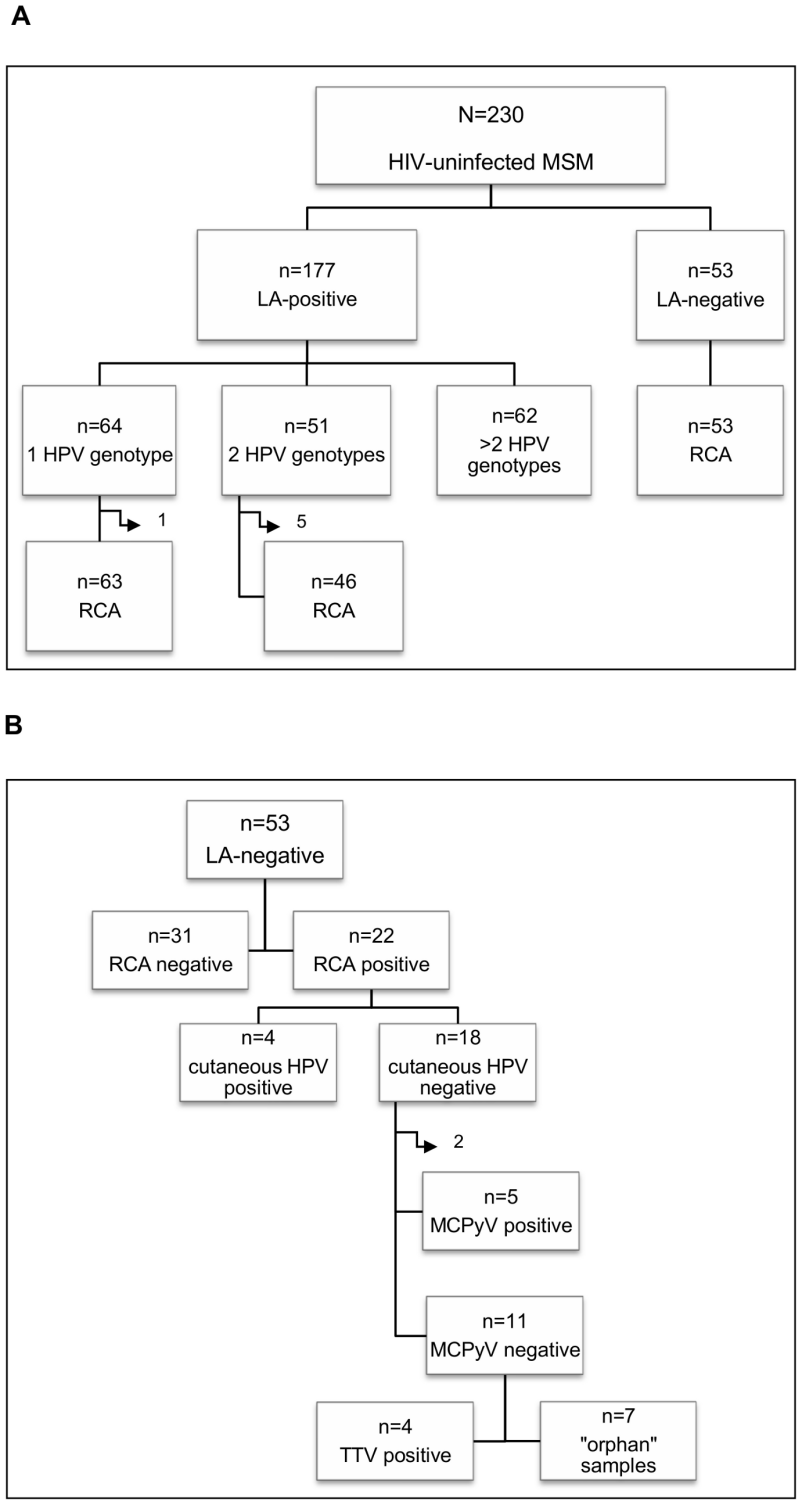
Genotype-specific prevalence of anal HPV infection. High-risk, possibly and probably high-risk types were considered as high-risk HPV, while low-risk and undetermined types were considered as low-risk HPV.

**Figure 2:**
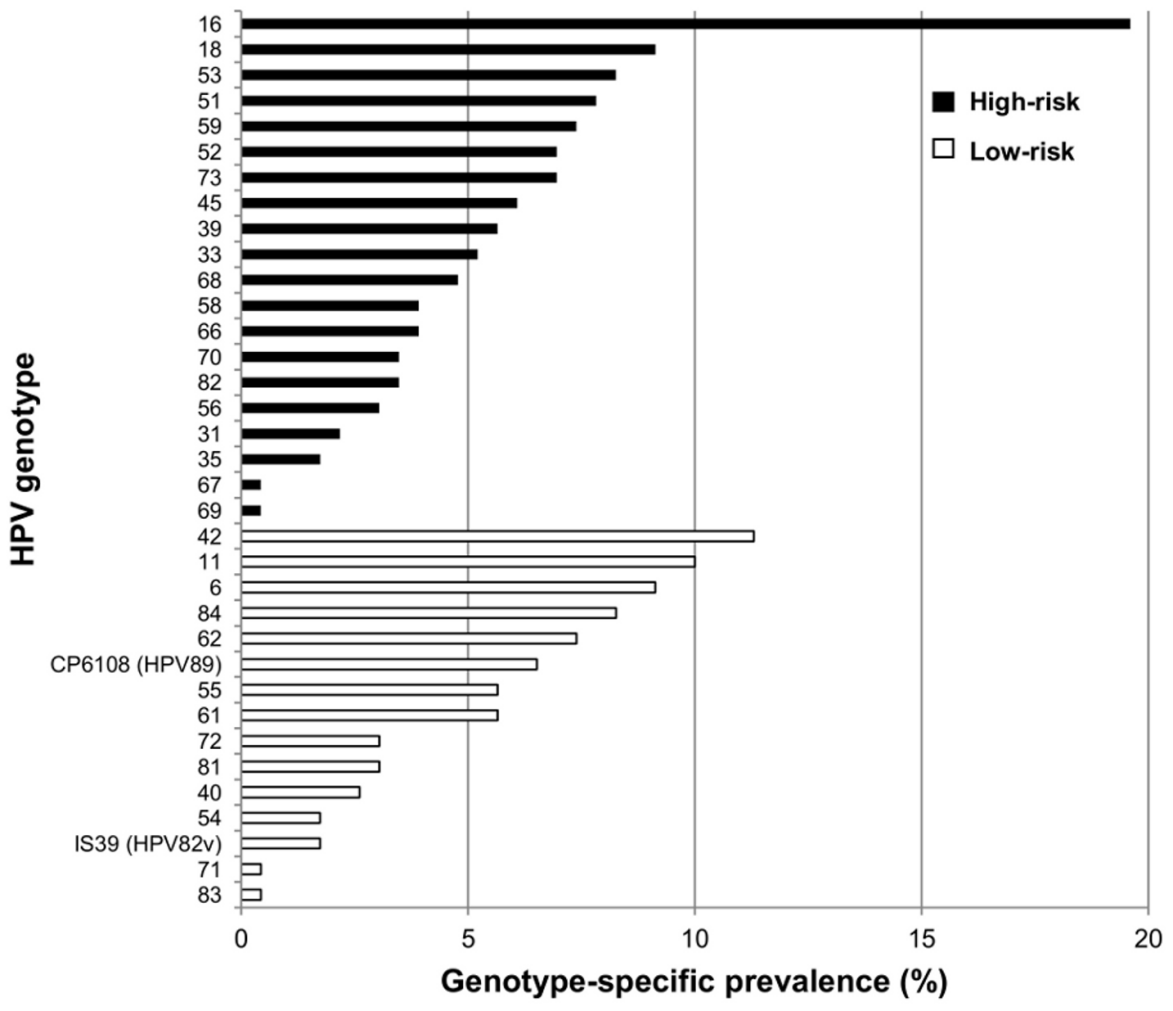
Schematic representation of the main molecular findings regarding the study samples. **A.** Summary of the Linear Array (LA) results and number of HPV genotypes detected in the LA-positive samples. Side arrows indicate samples excluded from the RCA analysis because of insufficient material. **B.** Summary of the RCA and virus-specific PCR results for the LA-negative anal samples.

Concerning anal HPV infection, 177 out of the 230 MSM (76.9%, 95% CI: 71.2–82.0) were positive: 64 (36.2%) were infected by a single HPV genotype, 51 (28.8%) by 2 genotypes and the remaining 62 (35.0%) by more than 2 genotypes (**Figure 1A**). A total of 35 different genotypes were found in our study population. The HPV genotype-specific prevalence is shown in **Figure 2**. Overall, HPV16 was the most common type (19.6%, 95% CI: 14.8–25.0). The most frequent low-risk type was HPV42, which was found in 11.3% (95% CI: 7.7–15.9) of the whole population.

**Figure 3:**
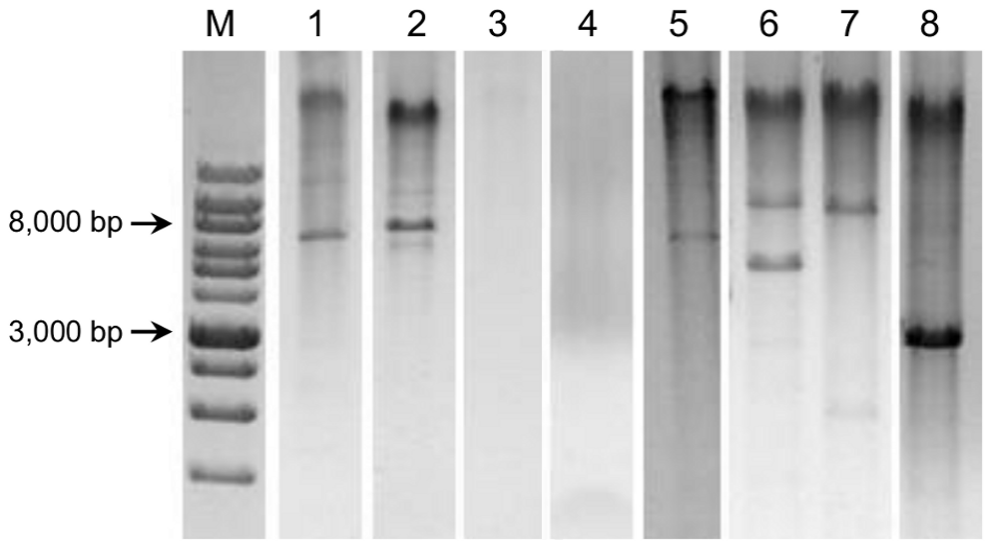
Restriction results of the Rolling Circle Amplification (RCA) products. Lanes: **1**. HPV6-positive sample digested with BamHI: the linearized genome of the expected size for the episomal DNA (7902 bp) is visible; **2**. HPV16-positive sample digested with BamHI: the linearized genome of the expected size for the episomal DNA (7905 bp) is visible; **3**. HPV16-positive sample digested with BamHI: no restriction fragments are observed, suggesting the integration of the viral genome; **4.** Linear Array-negative sample digested with BamHI: no restriction fragments are visible; **5**–**8.** Linear Array-negative samples digested with BamHI: one or more bands are detected, suggesting the presence of other circular DNA viruses. **M.** Molecular marker DM013-R500 (Gene-DireX, USA).

**Figure 4:**
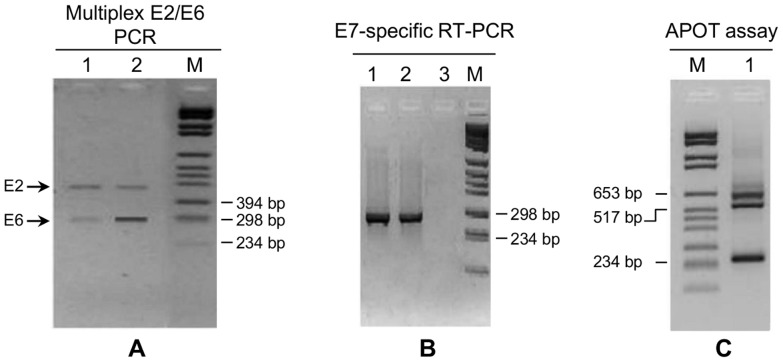
Analysis of HPV16 physical status in sample 132. **A.** Multiplex PCR for HPV16 E2/E6: 1. episomal infection (sample 74); 2. possible mixed infection suggested by a reduced E2 amplification with respect to E6 and an E2/E6 ratio <1.53 (sample 132). **B.** Amplification of E7-ecompassing cDNA using HPV16 E7-specific primers: 1. sample 132; 2. SiHa cells cDNA used as a positive control; 3. negative control (sterile water). **C.** APOT assay for the amplification of E7-encompassing viral cDNA using HPV16 E7-specific forward primer and (dT)17-p3 reverse primer: different amplicons were detected, none of them corresponding to the major E7-E1'E4 HPV16 episomal transcript of 1050 bp. **M**, molecular marker VI (Invitrogen).

Samples positive for 1 or 2 different HPV genotypes were subjected to RCA. Differently, the specimens containing more than 2 genotypes were excluded because of the anticipated complexity in the interpretation of the RCA restriction results, resulting in a loss of information for part of the HPV-positive samples. Six additional samples were excluded because the material was insufficient for the analysis. RCA was thus performed on a total of 109 mucosal HPV-positive specimens. Restriction analysis of the RCA products showed the expected fragments in all cases except one (sample 132), which was HPV16-positive (**Figure 3** **, lanes 1**–**3**). Thus, with the exception of one sample, all specimens harbored episomal viral DNA. The physical status of the viral genome in the 24 HPV16-positive samples was further analyzed by multiplex E2/E6 PCR. Apart from sample 132, the E2/E6 ratio of all the other HPV16-positive samples confirmed the episomal status. Differently, sample 132 E2/E6 ratio was <1.53, which would seem to suggest a possible mix of HPV16 episomal and integrated genomes (**Figure 4A**). Additionally, quantitative real-time PCR was performed on the 18 HPV16-positive samples with enough residual material. This assay confirmed the presence of pure episomal forms in all the cases except for sample 132, for which the ratio suggested a possible mixed form (**Table 1**). This analysis also evidenced a broad spectrum of viral genome copies, ranging from 2×10^1^ to 3.5×10^5^/ng of total DNA, similarly to other findings concerning this same anatomical site [27].

**Table 1:** qRT-PCR results, expressed as E2/E6 ratio, for HPV16-positive anal samples.

Sample n°	E2/E6 ratio	HPV16 physical status*
**116**	0.99	Episomal
**132**	0.40	Mixed
**162**	1.00	Episomal
**197**	1.00	Episomal
**200**	1.03	Episomal
**206**	1.01	Episomal
**207**	1.00	Episomal
**213**	0.98	Episomal
**228**	1.03	Episomal
**234**	1.00	Episomal
**248**	1.00	Episomal
**255**	1.14	Episomal
**278**	1.02	Episomal
**296**	0.96	Episomal
**352**	1.00	Episomal
**353**	1.11	Episomal
**382**	1.00	Episomal
**394**	1.05	Episomal

*inferred from the cut-off value for mixed versus episomal forms (0.60).

To investigate further the physical status of HPV16 genome in sample 132, the APOT assay, which allows to characterize HPV oncogene transcripts, was performed. Amplification of sample 132 cDNA with HPV16E7-specific primers evidenced E7-encompassing transcripts (**Figure 4B**). Through the APOT assay, three amplicons of different size were observed, although none of them displayed the size of the major E7-E1'E4 episomal HPV16 transcript, which is approximately 1050 bp (**Figure 4C**). Notably, the 250 bp fragment derived from misannealing of the (dT)17-p3 primer to an adenosine-rich sequence within HPV16 E1 gene, as reported in previous studies [13]. The other two amplicons, approximately 500 and 600 bp in size, were sequenced. Sequence analysis confirmed the integration and revealed a type B viral transcript, i.e., the use of the viral splice donor (SD880) and viral splice acceptor sites (SA3358). Moreover, it evidenced that the integration had occurred in chromosome 14 q. Therefore, the APOT assay indicated the exclusive presence of integrated genome-derived viral transcripts.

**Figure 5:**
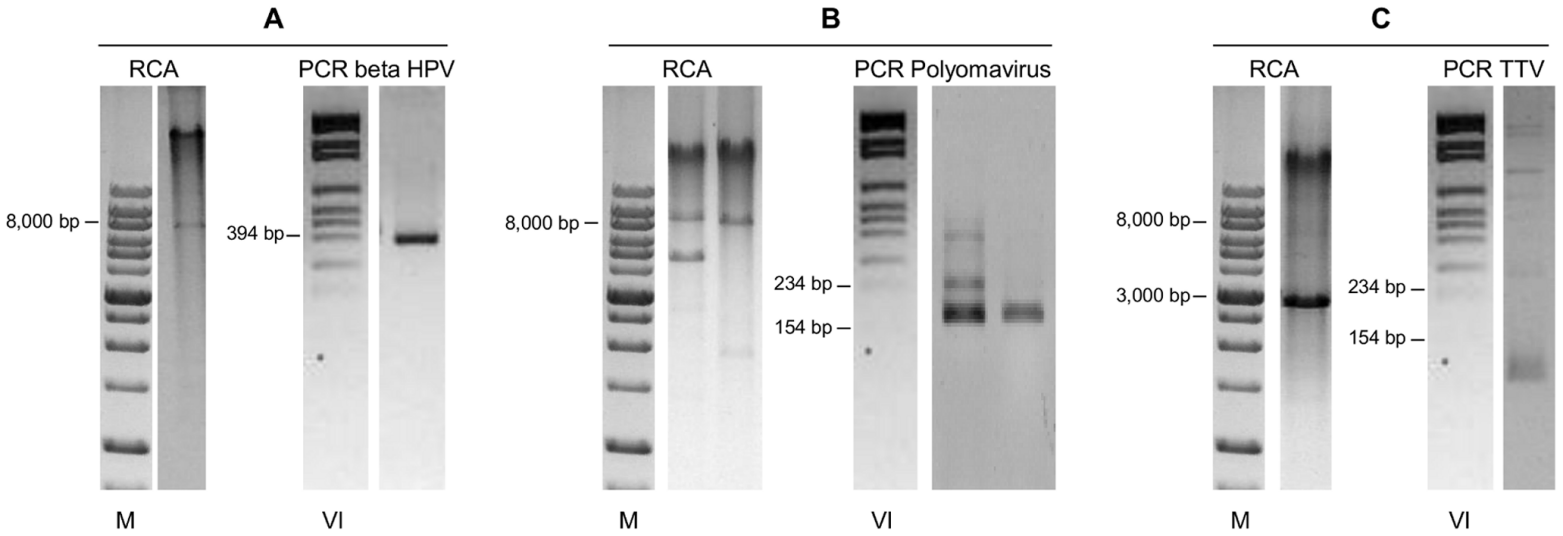
RCA analysis and specific PCR analysis of Linear Array-negative, RCA positive samples. **A.** RCA product digestion with Bam HI showed a band of about 8,000 bp; the sample was positive for cutaneous HPV following amplification with CP primers; **B.** RCA product digestion with Bam HI showed several restriction fragments ≤8,000 bp; these samples were negative for cutaneous HPV (not shown) but positive for Polyomavirus; **C.** RCA product digestion with Bam HI showed a band of about 3,000 bp; the sample was negative for cutaneous HPV and for Polyomavirus, but positive for TTV. Proper negative and positive controls were used for each virus-specific PCR (not shown). **M**, molecular marker DM013-R500 (Gene-DireX, USA). **VI**, molecular marker VI (Roche).

Fifty-three samples that tested negative by the Linear Array were subjected to RCA in order to explore the presence of viruses other than mucosal HPV (**Figure 1B**). In 31 samples (58.5%) no restriction fragments were observed after digestion of the RCA possible products with Bam HI (**Figure 3** **, lane 4**). Differently, the remaining 22 samples (41.5%) showed several restriction products (**Figure 3** **, lanes 5**–**8**). As these results would seem to suggest the presence of circular DNA molecules, possibly viral genomes, several attempts to characterize them were made. Given that contamination with cutaneous HPV genotypes from the perianal area is possible during anal sample collection, these 22 specimens were screened for cutaneous HPV genotypes. Four samples were positive (**Figure 5A**). With the exception of two samples that did not have enough material for further analysis, the other 20 were also tested for Polyomavirus, as it has been reported that these viruses are chronically shed from human skin and can be detected by RCA [28]. Importantly, they have already been found in anal samples of MSM [29]. Five of the samples analyzed showed a positive nPCR result (**Figure 5B**). Four of these samples matched MCPyV isolate 915F06008CG4 (GenBank accession number JQ479320), while one matched isolate 915F06007FD3 (GenBank accession number JQ479319) [30].

Finally, Bam HI restriction of RCA products evidenced in a few cases a clear band ranging in size between 2 and 3.5 Kb (**Figure 3** **, lane 8**), possibly deriving from small circular DNA viruses. Since TTV is an ubiquitous virus detected in multiple human tissues [25] and biological specimens, included saliva, cervical smears and feces [31], the size of which is compatible with the observed bands, the 11 “orphan” samples were subjected to a human TTV-specific PCR. Four of these samples showed a positive result (**Figure 5C**).

## Discussion

Anal HPV infection is very common in MSM, and is mainly associated with receptive anal intercourses and number of sexual partners. In a large series of HIV-uninfected MSM, we found an overall anal HPV prevalence of 76.9%, and confirmed that HPV16 is the most common type in anal infections of HIV-uninfected MSM [32], [33]. HPV42 was the most prevalent low-risk type, while HPV6 often represents the most common low-risk HPV in comparable studies [34], [35].

Since HPV infection is associated with the development of anal cancer and HPV-dependent carcinogenesis seems to be also linked to viral genome integration, we aimed to investigate this aspect in our sample series. Southern Blot is considered as the gold standard for the assessment of HPV physical status. Yet, this technique cannot be employed on limited quantities of DNA, such as those derived from anal brushing. There exist other sensitive methods that can be used to investigate HPV status, such as RS-PCR, DIPS PCR and APOT. However, RS-PCR and DIPS PCR are based on multiple amplifications for each sample, thus require large concentrations of DNA. Therefore, these methods can only be utilized when large amounts of clinical material are available and are impracticable with the limited amount of DNA recovered from anal cytological samples. APOT assay, which would allow distinguishing pure episomal and mixed forms of viral genome, may not detect viral-cellular fusion mRNA in the presence of an excess of episome-derived transcripts. In addition, it may not allow differentiating integrate-derived transcripts from transcripts derived from episomal HPV when the viral genome is integrated in form of a concatamer. Because of the abovementioned reasons, the RCA, which does not require large amounts of DNA and is feasible on a high number of samples, was used in this study. RCA with random hexamers was specifically chosen because of the secondary aim of investigating the presence of other DNA viruses apart from HPV. In fact, following its first application to viruses in 2004, phi29 polymerase-mediated RCA techniques have been developed in important tools for many applications in virology, including differentiation between linear and circular forms and detection of unknown viruses [36]. However, with respect to RCA with HPV specific degenerate primers, RCA with random hexamers presents the risk of background amplification of other confounders, e.g., genomic DNA of the same sample. Additionally, RCA does not allow to evidence directly the viral integration, which can only be hypothesized whenever no restriction fragments are observed upon RCA product restriction. At the same time, the presence of restriction fragments does not allow to exclude mixed forms of integrated and episomal DNA.

A previous study conducted in HIV-infected men showed anal HPV16 integration in 32% of the patients and evidenced that prevalence of the integration increased with the grade of cytological abnormalities, peaking in men with H-SIL [37]. Importantly, integration resulted the most significant risk factor for anal cytological abnormalities. In this study, none of the participants had high-grade lesions and HPV integration was only observed in one patient, who was positive for HPV16 and had a negative cytology report. HPV integration was initially suggested by the RCA results, and was then confirmed by the APOT assay that exclusively evidenced integrate-derived viral transcripts. Differently, both multiplex PCR and qRT-PCR suggested the presence of both episomal and integrated viral genomes. These data indicate that these assays may not always be suitable to discern between episomal and integrated forms. In fact, they would not be useful to identify integration events that do not involve E2 disruption or that occur in multiple tandem copies. The first case is well documented in cervical carcinoma SiHa cells, where integration occurred with a partial conservation of E2 gene [17]. In the latter case, E2/E6 ratio would fail to evidence the integration or would rather suggest the presence of mixed forms.

Interestingly, the pattern of the resolved amplicons obtained with APOT analysis on sample 132 resembled that found in both cervical cancer and in some HPV-positive head and neck cancers [38]. Sequencing of the amplification products revealed chimeric viral-cellular transcripts with integration in chromosome 14 q. This represents a transcriptionally active chromosomal region, reinforcing the hypothesis that viral integration may be facilitated in actively transcribed areas [39]. This analysis also clarified that the fusion transcript was of type B, i.e., it encompassed E7-E1 sequences at the 5′-end, followed by E4 and flanking cellular sequences at the 3'-end. Taken together, data from RCA and APOT suggested the mere presence of integrated genome in sample 132. However, in view of the multiplex PCR and qPCR results that evidenced E2 together with E6 amplification, E2 ORF seems to be partially retained, at least in the region of annealing of the specific primers used in these assays.

HPV genome integration seems to be promoted by several factors, among which infection persistence and viral replication in presence of DNA damage [9]. In this study the infection was only evaluated at baseline, thus no data about its persistence were available. However, clearance of anal HPV is common, with few individuals showing persistence unless HIV-infected [40]. Our data indicate that episomal forms are much more common than integrated ones in HIV-uninfected MSM, suggesting, although not proving, that clearance could be the most frequent event in our patients and that most of the observed infections were transient.

It is worth noting that the actual rate of integration may have been underestimated by the present study, since, as already underlined, the presence of integrated forms cannot be excluded in case of a positive RCA and more sensitive methods have only be used on HPV16-positive samples. Moreover, in this study HPV integration was not analyzed in histologically-confirmed anal lesions but only in cytological samples. However, the aim was not to ascertain HPV physical status in the lesions, but the frequency of integration in case of a detectable anal infection, irrespective of the presence of lesions. In addition, it has been shown that in case of HPV16 infection, APOT assay results obtained on cytological samples correspond almost completely to those obtained on biopsy material [13].

Among the Linear Array-negative anal samples, we found positivity for cutaneous HPV in four cases and for MCPyV in five cases. The presence of beta-HPV may be due to contamination from the skin of the perianal area during sample collection or from perianal plucked hairs, in which cutaneous HPV have been found [41]. Therefore, it is plausible that cutaneous HPV were present also in a certain proportion of the Linear Array-positive samples, although this possibility was not explored. Recently, it has been demonstrated a high prevalence of beta-HPV in external genital lesions and it has been postulated that certain genotypes, such as HPV38, may have a role in their development [42]. Yet, no data are available about a possible causative role of cutaneous HPV in anal lesions.

Mounting evidence supports the concept that MCPyV is a causal factor of Merkel cell carcinoma (MCC), a highly lethal form of skin cancer [43]. MCPyV has also been detected in healthy skin and mucosal samples of patients without MCC. Moreover, MCPyV has been found in 31% of anal, penile and oral samples of HIV-infected MSM [29]. For the first time, we report here the presence of MCPyV in anal samples of HIV-uninfected MSM without MCC. However, MCPyV role in the development of anal lesions seems unlikely. Indeed, Wieland et al showed that MCPyV anal positivity was higher in samples with normal cytology and benign lesions than in dysplastic/neoplastic specimens. Therefore, it is likely that MCPyV is prevalent in the general population, although its distribution might not be as widespread as that of cutaneous HPV.

This is the first report showing the presence of TTV in anal samples. The 2–3.5 Kb RCA restriction bands observed in four samples might correspond to full-length genomes and intragenomic rearranged TTV subviral molecules [44], which have already been demonstrated in other clinical samples [45]. So far, the ubiquitous nature of TTV (100% of 1 year-old infants are infected) has hampered efforts to associate it with the pathogenesis of a specific disease, although a possible etiological association with several conditions has been reported (e.g., of liver and respiratory tract). The presence of these anelliviruses in anal samples may reflect the high frequency of TTV infection in the general population, without any pathological significance.

In conclusion, RCA, together with E2/E6 multiplex PCR, qPCR and APOT assay used on HPV16-positive samples, did not evidence a high rate of anal HPV integration in HIV-uninfected MSM with no or mild cytological abnormalities. However, considering the limitations of the RCA and the use of more sensitive methods exclusively for HPV16-positive samples, the integration rate might have been underestimated. Importantly, RCA allowed to evidence that a variety of circular DNA viruses are present in the anal canal. Further studies are needed to ascertain the significance of these infections.
